# Interleukin-12 is not essential for silicosis in mice

**DOI:** 10.1186/1743-8977-3-2

**Published:** 2006-01-05

**Authors:** Gerald S Davis, Linda M Pfeiffer, David R Hemenway, Mercedes Rincon

**Affiliations:** 1 Pulmonary Disease & Critical Care Medicine Unit, Department of Medicine, College of Medicine, University of Vermont, Burlington, Vermont, USA; 2 Department of Civil & Environmental Engineering, College of Engineering & Mathematical Sciences, University of Vermont, Burlington, Vermont, USA; 3 Immunobiology Unit, Department of Medicine, College of Medicine, University of Vermont, Burlington, Vermont, USA

## Abstract

**Background:**

Silicosis features foci of inflammation where macrophages and lymphocytes precede and accompany fibroblast proliferation, alveolar epithelial hyperplasia, and increased deposition of connective tissue matrix material. In the mouse following silica inhalation there is recruitment of natural killer-, B-, and CD4^+ ^and CD8^+ ^lymphocytes to the alveolar spaces, enlargement of bronchial-associated lymphoid tissues (BALT), and aggregation of lymphocytes surrounding small airways and blood vessels. A substantial fraction of the recruited lung lymphocytes produce interferon-γ (IFN-γ), and IFN-γ gene-deleted mice develop less silicosis than wild-type mice. Interleukin-12 (IL-12) is an important pathway for driving the adaptive immune response towards a TH1-like phenotype. We hypothesized that IL-12 might stimulate lymphocyte activation and the up-regulation of IFN-γ, and consequently be an essential mediator for silicosis.

**Results:**

C57Bl/6 wild-type (WT) and IL-12 deficient (IL-12 KO) mice were exposed to sham-air or crystobalite silica (61 mg/m^3^) by inhalation for 5 hours/day for 12 days and then studied from 1 to 112 days after exposure. Mice exposed to sham-air had normal lung histology at all time points. WT mice exposed to titanium dioxide (72 mg/m^3^) showed pulmonary macrophage recruitment but no increase in lung collagen. Both WT and IL-12 KO mice exposed to silica showed similar progressive lung pathology, increased wet lung weight and increased total lung collagen (hydroxyproline). IL-12 p35 mRNA was not increased in either strain after silica exposure; IL-12 p40 mRNA was up-regulated after silica in WT mice and constitutively absent in the IL-12 KO mice. IL-18 mRNA was not increased after silica exposure. The expression of IL-15 (an important driver for innate immunity, Natural Killer cell activation, and IFN-γ production) was abundant in air-exposed mice and was increased slightly in the lungs of mice with silicosis.

**Conclusion:**

The axis of IL-12 driving IFN-γ production is not essential for the full manifestations of silicosis in mice exposed to a crystobalite silica aerosol.

## Background

Silicosis is a chronic diffuse parenchymal lung disease caused by the inhalation of respirable particles of crystalline silica. In the lung, foci of inflammation featuring macrophages and lymphocytes precede and accompany fibroblast proliferation, alveolar epithelial hyperplasia, and increased deposition of connective tissue matrix material. The mechanisms through which silica triggers these responses have been clarified over the past four decades, but many key pathways remain unknown [1-7]. We have used mice exposed to silica by inhalation as a test system to elucidate some of these pathways [8]. In particular, we have focused on cytokines produced by macrophages that may recruit and activate lymphocytes and fibroblasts, and cytokines produced by lymphocytes that may in turn activate macrophages and modify fibroblast function [9-14].

Lymphocytes are a prominent feature of the lung lesions of silicosis, both in man and in experimental rodents. In the mouse following silica inhalation there is prompt and persistent recruitment of lymphocytes to the alveolar spaces, enlargement of bronchial-associated lymphoid tissues (BALT), and aggregation of lymphocytes surrounding small airways and blood vessels [14]. These recruited lung lymphocytes include natural killer (NK) cells, B-cells, CD4^+ ^T-cells, and CD8^+ ^T-cells in greatly increased numbers but in proportions similar to those in the normal mouse lung [12,15].

A substantial fraction of the recruited lung lymphocytes in murine silicosis produce interferon-γ (IFN-γ). The numbers of cells containing IFN-γ protein, the abundance of mRNA for IFN-γ, and the frequency of sites with cells containing mRNA for IFN-γ *in situ *are increased [11]. Conversely, the abundance of interleukin-4 (IL-4) appears to be relatively decreased in this inhalation model system. Mice that constitutively lack IFN-γ production (C57Bl/6-*Ifng*^*1Ts*^) develop less extensive lung pathology and less lung collagen deposition early after silica inhalation [14]. These observations suggest that silicosis resembles the T_H_1 type of response described for the adaptive immune response, or a similar T_H_1-like response that is important for the innate response. The exact roles for IFN-γ remain uncertain, since this cytokine could act early in the response to silica to recruit and activate macrophages and lymphocytes [16,17]. IFN-γ might also act later in silicosis to down-regulate fibroblast responses to transforming growth factor-β (TGF-β) and decrease collagen production [18-22].

Interleukin-12 (IL-12) is an important macrophage-derived cytokine that can drive IFN-γ production [23]. The mature biologically active IL-12 protein is a heterodimer composed of a p35 subunit and a p40 subunit that are assembled to produce the secreted p70 form. An increase in IL-12 is a prime determinant that biases uncommitted (T_H_0) lymphocytes towards a T_H_1-type response in antigen-driven adaptive immunity, and IL-12 is an essential cytokine for an effective response to intracellular microbial pathogens. Monomers or dimers (p80) of the IL-12 p40 peptide have distinct activities. Interleukin-18 augments but cannot replace the actions of IL-12 on IFN-γ production [24-26].

Interleukin-15 (IL-15) is produced in the bone marrow and by a variety of lymphoid and mesenchymal cells in peripheral organs. IL-15 is critical for the bone marrow proliferation of NK cells and for the peripheral organ activation of NK cells [27-31]. IL-15 offers an alternative stimulus which can up-regulate IFN-γ production in NK cells and T-cells, and appears to be a key cytokine in the innate immune response.

We hypothesized that silica might stimulate macrophages to produce IL-12, and possibly IL-18, and that these cytokines would be essential factors in generating IFN-γ. We postulated that when mice lacking the ability to produce IL-12 were exposed to silica they would develop less IFN-γ production, less mononuclear cell inflammation, and less lung collagen accumulation than wild-type IL-12-sufficient mice. We tested this hypothesis by exposing to silica a strain of genetically modified mice wherein the IL-12 p40 gene had been deleted (IL-12 KO; B6.129S1-*Il12b *^*tm1jm*^) [32,33]. The hypothesis proved to be incorrect. The IL-12 KO mice and the C57Bl/6 wild-type mice exposed to silica developed comparable lung pathology, lung collagen accumulation, and up-regulation of IFN-γ mRNA expression. Mice exposed to sham-air or to the inert particle titanium dioxide (TiO_2_) had normal lungs and served as comparisons for the silica-exposed mice. Thus IL-12 signaling was not essential in silicosis and alternative pathways to a T_H_1-like innate response may be more important.

## Results

### Growth and general health

All mice from both C57Bl/6 and IL-12 KO strains appeared to be healthy and showed normal behavior regardless of exposure to sham-air or inhalation of cristobalite silica or titanium dioxide for 5 hours per day for 12 days. There were no unplanned deaths among the mice. All of the mice gained weight normally and similarly; mice at 1 day post-exposure averaged 22.4 g ± 2.2 g (mean ± SD) and mice at 112 days weighed 31.4 g ± 2.3 g, a 40% weight gain, with no differences among groups at any time point.

### Lung pathology

**Figure 1:**
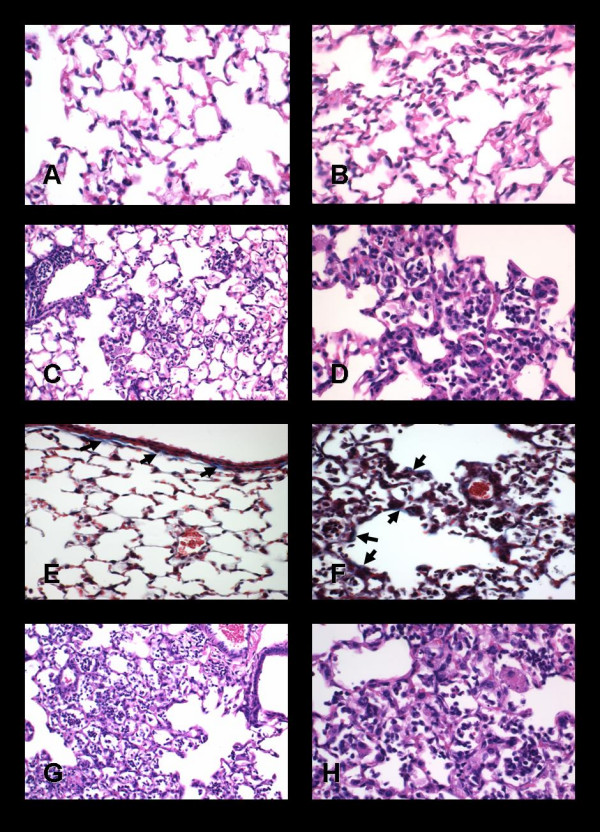
**Pathology in wild-type and IL-12 knock-out Mice**. This figure illustrates the lung pathology of C57Bl/6 wild-type and IL-12 KO mice 112 days after inhalation exposure to sham-air, rutile titanium dioxide, or cristobalite silica. A lung section from an air-exposed mouse shows normal histology (**A**, H&E, 400×). A lung section from a mouse exposed to TiO_2 _(**B**, H&E, 400×) shows increased numbers of macrophages, and many of these cells are laden with particulates. No inflammatory foci are present, and the alveolar walls appear normal. Tissue from wild-type mice exposed to silica (**C**, H&E, 200×; **D**, H&E, 400×) show numerous patchy collections of macrophages and lymphocytes, with apparent hyperplastic alveolar epithelial cells and fibroblasts. Cuffs of lymphocytes surround some arterioles and airways. Trichrome stain reveals bright blue connective tissue matrix and collagen (arrows) beneath the epithelium of the bronchial wall in a section from a sham-air exposed mouse (**E**), but rare matrix staining in the alveolar septae (400×). A silica-exposed lung (**F**) shows bright blue trichrome stained matrix in alveolar walls (arrows) within a silicotic lesion (400×). Sections from silica-exposed IL-12 KO mice show typical features of silicosis (**G**, H&E, 200×; **H**, H&E, 400×). The extent and features of disease are as intense as those found in the wild-type silica-exposed mice.

Wild-type C57Bl/6 mice exposed to sham-air had lungs which demonstrated normal histology at all time points throughout the 16 weeks of study; a representative lung section is shown in Figure 1A. Trichrome stain revealed bright blue collagen fibers in the adventitia surrounding larger blood vessels and airways, delicate bands of collagen beneath bronchial epithelium (see Figure 1E) and in the pleura, and occasional thin strands within the alveolar walls. The IL-12 KO mice exposed to sham-air also had normal lung histology at all time points. Samples of the spleen and the thymus were collected from all mice, and appeared histologically normal in all cases. The mediastinal/right paratracheal lymph nodes from mice exposed to sham-air appeared normal in size and structure.

The effects of exposure to TiO_2 _were minimal, but detectable at all time points. Alveolar macrophages laden with particles were notable at day 1 and appeared to be increased in number. These cells were evident at 14, 42, and 112 days as well. Other than increased numbers of macrophages in the airspaces, the lungs of TiO_2_-exposed mice appeared to be normal. The alveolar walls were not thickened, collections of lymphocytes were not found, and foci of fibroblasts or hypertrophic Type II alveolar epithelial cells were not apparent. A representative section from the lung of a mouse exposed to TiO_2 _112 days previously is shown in Figure 1B.

Inhalation of cristobalite silica caused pathological responses that were evident by day 14 but not apparent as early as day 1 in both the wild-type and IL-12 KO mice. In contrast to the TiO_2 _exposure, increased numbers of macrophages were not found 1 day after ending the 12 days of exposure. The cristobalite particles are not birefringent or visible by polarized light microscopy (as is quartz), thus they cannot be seen readily by light microscopy. By 14 days post-exposure to silica the wild-type mice demonstrated collections of inflammatory cells, scattered alveolar exudates, and areas of hyperplastic Type II epithelial cells. At 42 days after exposure more extensive lung tissue changes, collections of lymphocytes surrounding small airways and arterioles, and loose aggregates of fibroblast-like cells were evident. These pathological features were more pronounced by 112 days after exposure, and small foci of increased connective tissue matrix material could also be found (see Figure 1F). The lung pathology at 112 days is illustrated in Figures 1C and 1D. Trichrome stain revealed bright blue bands of collagen fibers in alveolar walls in silicotic lesions (Figure 1F). The silica-exposed IL-12 KO mice at all time points demonstrated pathological changes that were similar in quality and extent to those found in the C57Bl/6 wild-type mice. The pathology was, if anything, more extensive in the IL-12 KO mice than in the wild-type animals, as shown in Figures 1G and 1H.

The mediastinal/right paratracheal lymph nodes from mice exposed to silica were greatly enlarged at 42 and 112 days after exposure in both the wild-type and IL-12 KO mice. The microscopic pathology showed enlargement of the germinal centers and the cortex, and increased numbers of germinal centers, as we have reported previously in rodents [10,13].

**Figure 2:**
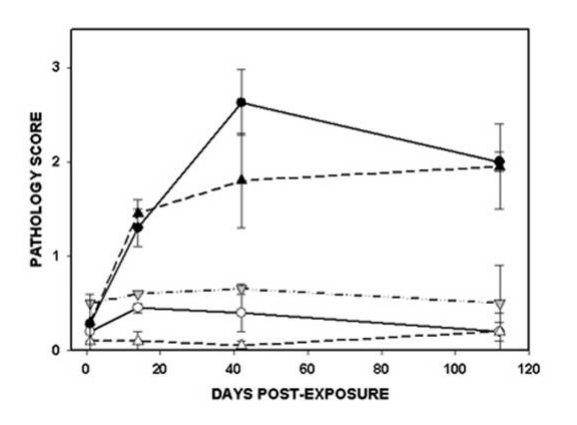
**Lung pathology score**. The extent of abnormality was graded semi-quantitatively in 10 areas of lung sections from 2 mice in from each strain and exposure group at four time points. The data are shown as the average value, with individual scores shown as the range bar. The significance of differences among the groups is listed in the text.

The lung tissue sections from all mice (N = 40) were graded for the extent of silicosis as described in Table 1. Seventeen slides were graded twice in order to assess observer reproducibility in scoring. No specimens in this experiment averaged a score above 3.0, while previous studies have shown occasional samples scoring as high as 4.0. Most of the slides scored twice had a difference of 0.5 or less between the first and second score (r = 0.62). The pathology scores from the wild-type and the IL-12 KO mice are shown in Figure 2. All of the lung sections from C57Bl/6 mice exposed to sham air were scored as 0.6 or less, and the air-exposed IL-12 KO mice were scored as 0.3 or less. The mice exposed to TiO_2 _scored 0.9 or less, although they were recognizable because of particle-laden macrophages. At 1 day post-exposure the wild-type mice exposed to silica scored normally (0.3) and were indistinguishable from the sham-air specimens. Scores greater than 1.0 were recorded for all silica-exposed samples after 1 day. The IL-12 KO mice exposed to silica were scored as normal at 1 day, but had abnormal scores at all subsequent time points. The wild-type C57Bl/6 mice exposed to silica scored significantly (ANOVA p < 0.05) higher than air- or TiO_2_-exposed mice at 14, 42, and 112 days. The silica-exposed IL-12 KO mice scored significantly higher than their air-exposed litter mates at day 14, and the differences approached significance (0.050 < p < 0.070) at days 42 and 112. The wild-type and the IL-12 KO mice exposed to silica tracked closely at all time points, and there were no significant differences between them.

**Table 1:** Grading scale for silicosis in mice

**Grade**	**Degree**	**Description**
0	Normal histology	Delicate alveolar walls, without inflammatory cells or alveolar exudates.
1	Minimal changes	Increased numbers of macrophages, rare inflammatory cell collections, normal alveolar walls
2	Slight silicosis	Occasional foci of macrophages and inflammatory cells, scattered areas of alveolar exudates, rare peribronchial and perivascular accumulations of lymphocytes.
3	Moderate silicosis	Frequent foci with alveolar wall thickening, alveolar exudates, accumulations of inflammatory cells; hyperplastic Type II alveolar epithelial cells and collections of fibroblasts may be seen; some areas of lung may appear nearly normal.
4	Severe silicosis	Almost the entire lung is obviously abnormal; numerous large accumulations of inflammatory cells, peribronchial and perivascular lymphocytes, alveolar remodeling.

Tissue sections were examined at 200× magnification and 10 microscopic fields were graded to generate an average score for each mouse.

### Lung wet weight

**Figure 3:**
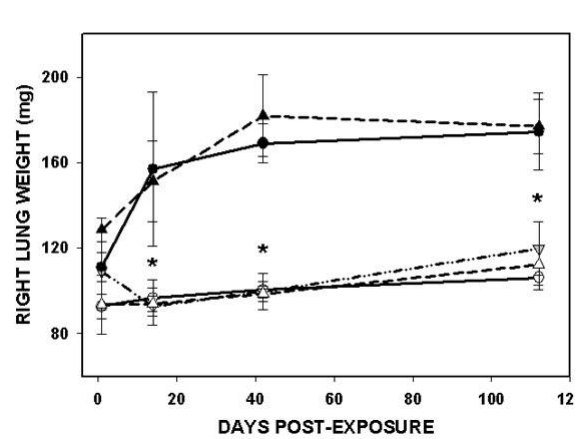
**Right lung wet weight**. The wet weights (mg) of the right lungs from 5 mice in each strain and exposure group are shown at four time points after exposure. The data are shown as mean ± SD for each group. The wild-type and IL-12 KO mice exposed to silica were significantly different (* = p < 0.002) from the sham-air exposed mice. There were no significant differences among the mice exposed to air or TiO_2 _at any time point, or between the wild-type and IL-12 KO mice exposed to silica at any time point

The wet weight served as a crude indicator of the accumulated mass of connective tissue matrix material, structural cells, inflammatory cells, and edema fluid in the lung. The right lung from each mouse was excised for collagen analysis, and was weighed immediately after removal from the thorax. The values for right lung wet weight for each strain and exposure group at the study time points are shown in Figure 3. The wet weight of the right lung in sham-air exposed wild-type mice increased over the 16 weeks of observation (92.8 mg to 106.2 mg), a 14% increase compared to a 40% increase in total body weight. The right lung weights increased similarly in wild-type mice exposed to sham-air or TiO_2 _and in IL-12 KO mice exposed to sham-air. There were no significant differences among the air or TiO_2_groups at any time after exposure.

The lungs of mice exposed to silica were increased in weight at all time points. One day after the exposure ended the lung wet weights from both wild-type and IL-12 KO mice were increased slightly, but the differences did not reach significance. At days 14, 42, and 112 both silica-exposed groups showed lung weights that were significantly increased, and were approximately 65% greater than the sham-air controls. There were no significant differences between the silica-exposed wild-type and the IL-12 KO mice at any time after exposure.

### Lung collagen

**Figure 4:**
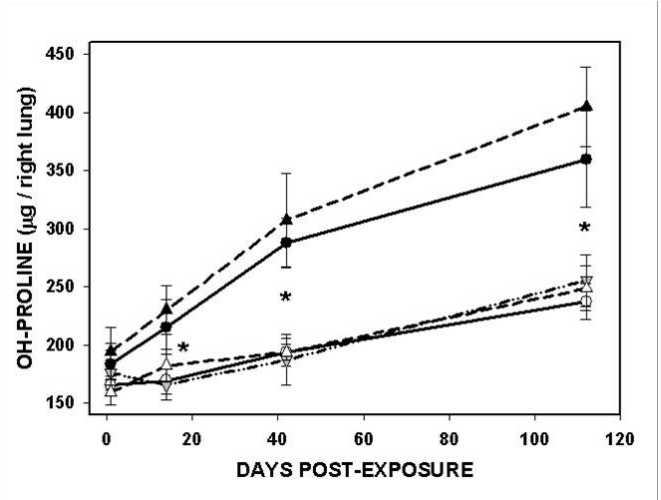
**Total lung collagen**. The accumulation of collagen in the lung was assessed as the amount of OH-proline per right lung, with 5 mice in each group for each strain, exposure, and time point. The C57Bl/6 wild-type and the IL-12 KO mice exposed to silica had significantly more OH-Proline per right lung than the air- or TiO_2_-exposed mice at 14, 42, and 112 days after exposure (* = p < 0.01). The differences between the IL-12 KO mice and the wild-type mice exposed to silica did not reach significance.

The extent of pulmonary fibrosis was reflected by measuring total lung collagen, assessed biochemically as the quantity of hydroxyproline (OH-Pro) in the right lung. The results from 5 mice in each group are shown in Figure 4. The amount of OH-Pro was greater in the lungs of silica-exposed mice at all time points after exposure, and the differences reached significance at 14, 42, and 112 days. There were no differences among the wild-type air- exposed, wild-type TiO_2_-exposed, or the IL-12 KO air-exposed mice at any time points. The IL-12 KO mice exposed to silica had more lung OH-Pro at all four time points. Thus silica exposure caused as much or more excess collagen accumulation in the lungs of the IL-12 KO mice as in the wild-type animals.

### Cytokine gene expression

**Figure 5:**
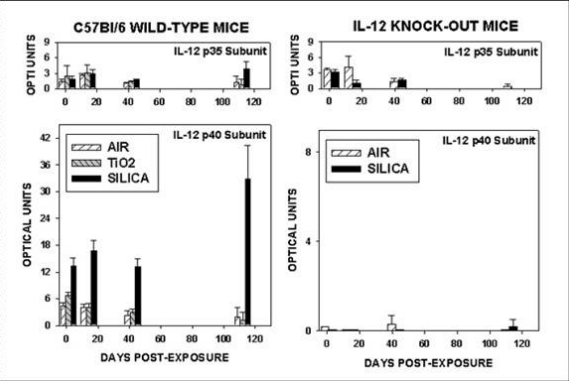
**Interleukin-12 gene expression**. The intensity of gene expression for both components of the IL-12 heterodimer was measured by ribonuclease protection assay in lung tissue samples from 3 mice from each strain and exposure condition. The results for C57Bl/6 wild-type mice are shown in the left panel and results for the IL-12 KO mice are shown in the right panel. The analysis for the IL-12 p35 subunit is shown in the upper panels and for the p40 subunit in the lower panels. The significance of the differences among the groups is presented in the text. The IL-12 p40 subunit mRNA was virtually undetectable in the IL-12 KO mice because this is the gene that has been modified to create the knock-out strain.

The intensity of gene expression for several cytokines of interest was measured by ribonuclease protection assay. Lung tissue samples from 3 mice from each strain and exposure were analyzed at 1, 14, 42 and 112 days after exposure. All of the samples and genes for each day were measured simultaneously in a single hybridization, digestion, and gel separation assay, and the results were adjusted for the abundance of two constitutively expressed genes (GAPDH, L32). The results for IL-12 p35 and IL-12 p40 gene expression are shown in Figure 5. IL-12 p35 was expressed in low abundance at all time points by both strains regardless of exposure history. There were no significant differences among the various groups.

The IL-12 p40 subunit was expressed at low and consistent levels by wild-type C57Bl/6 mice exposed to sham-air or to TiO_2 _at all time points, with no differences due to time or exposure. The wild-type mice exposed to silica showed significant elevation of IL-12 p40 expression (p < 0.002) at all four time points, with greatest abundance at 112 days post-exposure. The IL-12 p40 subunit mRNA was virtually undetectable in the IL-12 KO mice, as would be expected since this is the gene that was modified to create the strain [33].

**Figure 6:**
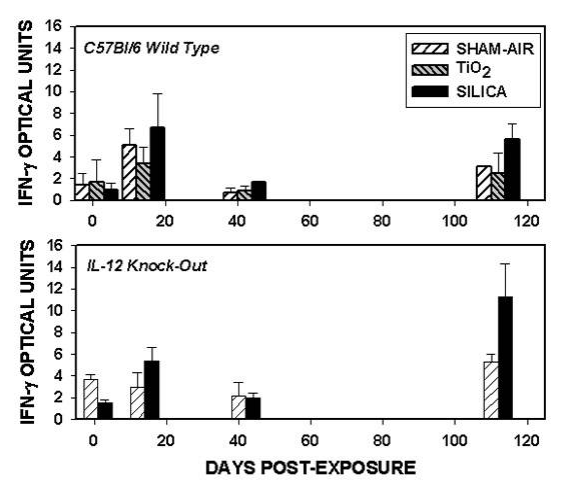
**Interferon-γ gene expression**. The abundance of mRNA for interferon-γ (IFN-γ) is shown for the wild-type (upper panel) and IL-12 gene-deleted (lower panel) mice.

The abundance of mRNA for interferon-γ (IFN-γ) is shown in Figure 6. In both wild-type and IL-12 KO mice the expression of IFN-γ appeared to be increased beyond 14 days after silica exposure compared to sham-air or TiO_2_-exposed mice, and approached but did not reach significance (0.05 < p < 0.070) at 112 days. There were no significant differences between the two strains; the IL-12 KO mice appeared to up-regulate IFN-γ gene expression despite the constitutive absence of IL-12 signaling.

**Figure 7:**
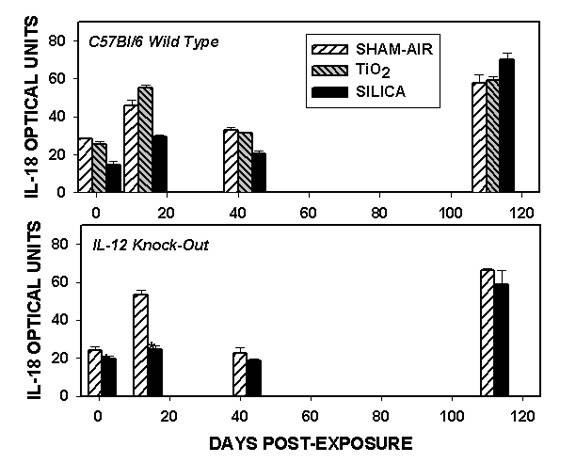
**Interleukin-18 gene expression**. The abundance of mRNA for IL-18 is shown for the wild-type (upper panel) and the IL-12 gene-deleted (lower panel) mice. The expression of IL-18 was significantly decreased at earlier time points after silica exposure in both strains (* = p < 0.01), but normalized by 112 days.

We examined the expression of the interleukin-18 (IL-18) gene, because this cytokine is known to augment the effects of IL-12 in stimulating IFN-γ production [24,26]. The abundance of mRNA for IL-18 is shown in Figure 7 for both wild-type and IL-12 KO mice. Message for this cytokine was abundant in both strains of mice exposed to sham-air or to TiO_2 _at all time points. The expression of IL-18 mRNA was significantly decreased in mice exposed to silica at the earlier time points (1 – 42 days), but was normalized in C57Bl/6 mice 112 days after exposure. There were no significant differences in IL-18 expression between silica-exposed wild-type and IL-12 KO mice at any time point.

**Figure 8:**
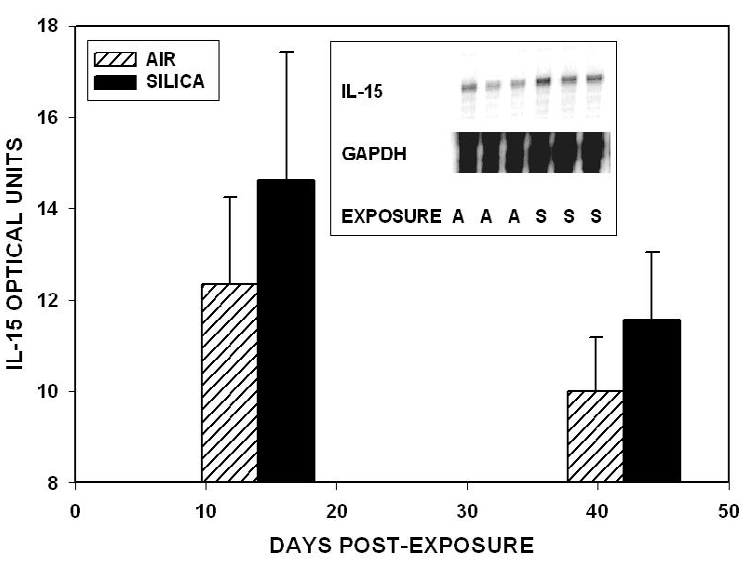
**Interleukin-15 gene expression**. The expression of IL-15 was measured in C57Bl/6 mice 14 and 42 days after exposure to sham-air or cristobalite silica. Data are shown as mean ± SD for 3 samples per group. The inset from 14 days post-exposure shows the polyacrilamide gel bands for IL-15 mRNA transcripts and the comparison ubiquitous gene GAPDH from the air- (**A**) and silica- (**S**) exposed lungs.

IL-15 is an alternative cytokine produced in the lung that could drive NK cell activation and up-regulate IFN-γ production. IL-15 expression was tested in separate similar experiments in which wild-type C57Bl/6 were exposed to sham-air or cristobalite silica for 5 hours per day for 12 days. As shown in Figure 8, the mRNA expression of IL-15 was abundant in air-exposed mice and was increased slightly in the lungs of mice exposed to silica at both 14 and 42 days.

## Discussion

### Silicosis in mice

The C57Bl/6 wild-type mice exposed to crystobalite silica developed pathological features of silicosis and progressively increased total lung collagen that were closely similar to those we have reported previously in C3H/HeN mice [8]. The IL-12 KO mice, also bred against a C57Bl/6 background, developed silicosis that was not distinguishable from that found in the wild-type animals. The mice of both strains that were exposed to sham-air appeared to be normal and had normal lung histology throughout the experiment. We used exposure to rutile TiO_2 _as a comparison for silica with a mineral particle expected to be biologically relatively inactive and not cause pulmonary fibrosis. Although the TiO_2 _did cause an increase in the number of macrophages observed in the lung, and numerous particles could be found in these cells, it did not trigger recruitment of substantial numbers of lymphocytes, neutrophils, or other cell types, and it did not cause an increase in lung collagen. These findings highlight the inflammatory and fibrotic properties of silica compared to other mineral particles, and demonstrate the specific features of the response to inhaled silica.

### IFN-γ regulation in silicosis

Interferon-gamma (IFN-γ) is a potent pleomorphic cytokine produced by activated T-lymphocytes, NK cells, macrophages and possibly other cell types [34,35]. The production of interferon-gamma (IFNgamma) in response to infection is a hallmark of innate and adaptive immunity, and its excessive release has been associated with the pathogenesis of chronic inflammatory and autoimmune diseases. Alterations in the abundance of IFN-γ have been reported in silicosis by several investigators using inhalation or intra-tracheal (IT) instillation in mice and rats, and in silica-exposed human workers.

Following inhalation exposure of C3H/HeN male mice we observed increased mRNA for IFN-γ, localization of IFN-γ mRNA transcripts to silicotic lung lesions and bronchial-associated lymphoid tissues (BALT), and increased numbers of lymphocytes (natural killer (NK) cells, CD4+ and CD8+) producing IFN-γ protein [11,12]. In the present study we observed expression of IFN-γ mRNA at 14 and 112 days, but not immediately (1 day), post exposure in both wild-type and IL-12 KO mice (Figure 6). Hubbard and colleagues [36] performed IT instillation of 1 mg quartz into NFκB-luciferase reporter mice, and studied the responses at times up to 3 days after exposure. NFκB was sharply increased in bronchial epithelial cells and macrophages; lung homogenate mRNA analysis showed increased IFN-γ and IL-12 (p40) expression in response to silica. Brown et al did not observe an increase in serum IFN-γ or IL-12 proteins in NZM mice treated by intra-nasal instillation with quartz, but did not measure lung or BAL cytokine levels [37]. Garn and associates examined the mediastinal lymph nodes of rats following silica inhalation and observed node hypertrophy with a large increase in the numbers of NK cells, CD4+ and CD8+ lymphocytes and macrophages [10]. The level of IFN-γ and IL-12 mRNA were increased, while IL-4 mRNA was not increased. Co-cultivation of macrophages and lymphocytes from silicotic nodes showed increased IFN-γ production [13]. Conversely, Huaux and colleagues have observed unchanged or decreased levels of IFN-γ mRNA in mice instilled IT with quartz [15,38-40]. Increased serum and urine levels of neopterin (a pteridine compound produced by human monocytes/macrophages upon stimulation with IFN-γ [7001]) have been detected in silica-exposed workers [41], implying up-regulation of IFN-γ in human silicosis.

The abrogation of IFN-γ signaling has had varying effects on silicosis in mice. When we exposed B6 mice to cristobalite silica inhalation and examined them 90 days later, IFN-γ gene-deleted (KO) mice developed significantly less extensive pathological changes and less collagen accumulation than wild type mice [14]. Desaki et al performed IT instillation of 2 mg of quartz into wild-type and IFN-γ KO mice [42]. At 21 days after instillation, similar inflammatory cell recruitment, lung pathology, and increased lung OH-proline were found in both strains. Chen and colleagues treated rats by IT instillation of 20 mg of quartz and then with daily intramuscular injections of human recombinant IFN-γ [41]. At 1 or 2 months after silica exposure the IFN-γ-treated rats showed lung pathology and collagen accumulation that was intermediate between saline-exposed and silica-exposed sham-cytokine animals, implying an anti-fibrotic effect from exogenous IFN-γ treatment.

### Interleukin-4 regulation in silicosis

Changes in IL-4 have been observed by some, but not all, investigators studying silicosis. Huaux and associates observed increased abundance of IL-4 signaling following IT instillation of quartz [15,38-40] and modulation of both the pathology and collagen accumulation in animals where IL-4 abundance was increased or decreased in association with over-expression of IL-10 or IL-9 respectively. Conversely, we did not find increased IL-4 mRNA or an increase in the percentage of lymphocytes producing IL-4 in mice exposed to cristobalite by inhalation [11,12]. Garn and colleagues noted decreased IL-4 mRNA in the lymph nodes of rats with silicosis [10,37]. Chen et al found increased IL-4 mRNA *in situ *after silica instillation into rats [43]. Desaki et al performed IT instillation of quartz into wild-type and genetically modified mice that lacked IL-4 (KO) [42]. Notably, the IL-4 deficient mice did not show any reduction in the extent of lung pathology or the accumulation of collagen.

### IL-12 Biology

Interleukin-12, initially described as "cytotoxic lymphocyte maturation factor" or "NK cell stimulatory factor", is a heterodimeric cytokine that can act as a growth factor for activated T-cells and NK-cells and can stimulate the production of IFN-γ by resting mononuclear cells [44,45]. The mature, biologically active secreted 70-kD (p70) cytokine is produced by the covalent cross-linking and glycosylation of two precursor chains encoded by genes on separate chromosomes, the p35 and p40 subunits. In mice, the p35 subunit is expressed in many lymphoid and non-lymphoid tissues including the lung, while the p40 subunit is expressed in lymphoid cells [44] and in airway epithelium [46]. The IL-12 related cytokines consist of glycoproteins encoded by 5 independently regulated genes that combine as hetero- or homodimers to produce 7 secreted proteins: IL-12 (the p40 and p35 heterodimer), p80 (a p40 homodimer), IL-23 (a p40 and p19 heterodimer), p40 monomer, and others [47]. Thus deletion of the *p40 *gene will affect all of these molecules.

Biologically active IL-12 is produced predominantly by macrophages, monocytes, and B-lymphocytes. Human alveolar macrophages secrete IL-12 in response to stimulation by endotoxin, IFN-γ, and other signals, with autocrine suppression by IL-10 [48]. IL-12 is produced promptly during the induction of the adaptive cell-mediated immune response to antigens and in response to intracellular pathogens such as *Myobacterium tuberculosis *or *Listeria monocytogenes*. Abundant IL-12 is an important signal which biases the antigen-driven adaptive immune response towards a T_H_1 (IFN-γ dominated) phenotype rather than a T_H_2 (IL-4 dominated) phenotype [45,49,50]. IL-12 drives target cells through a single high-affinity receptor composed of two subunits [23]. Uncommitted (T_H_0) T-cells and NK-cells appear to be the main targets for IL-12.

### Effects of IL-12 modulation

The biological functions of IL-12 have been tested in systems where the cytokine was augmented by injection, blocked with antibody, removed by modification of the p35 or p40 genes, or blocked by modification of the IL-12 receptor complex. In susceptible mouse strains treatment with exogenous recombinant murine IL-12 reduced tuberculosis mortality [51], delayed mammary tumor onset with reduced tumor burden [52], and inhibited hepatitis B virus infection [53], apparently through the actions of IFN-γ. Administration of IL-12 at the time of a single antigen challenge abolished airway hyper-responsiveness and pulmonary eosinophilia, and promoted increased IFN-γ and decreased IL-4 and IL-5 expression, in mice sensitized for asthma with ovalbumin [54].

Neutralization of IL-12 by the administration of antibody greatly increased susceptibility of mice to primary infection with *L. monocytogenes *[55], protected BCG-primed mice against a lethal response to endotoxin [56], and reduced experimental immune colitis [57]. The IL-12 p40 gene-deleted strain (IL-12 KO used for this report) developed by Magram and colleagues [33] showed increased susceptibility to infection with *M. tuberculosis *[58], *Cryptococcus neoformans *[59], and *Leishmania major *[60], and showed diminished severity of collagen-induced arthritis [61]. Wild-type C57BL/6 mice developed self-resolving *Leishmania chagasi *infection with abundant IFN-gamma; in contrast, *L. chagasi *disease was exacerbated and IFN-gamma was low in IL-12KO mice [62].

### Discoordinated production of IL-12 p40

The *p40 *gene subunit of IL-12 may be expressed and the peptide product translated at high levels without coordinate expression of the *p35 *subunit or production of the complete p70 cytokine. Potentially, these products could function as p80 homodimer or p40 monomer. In this report we observed increased levels of p40 transcripts with no change in the abundance of p35 levels in mice exposed to silica, as shown in Figure 5.

Soluble p40 peptide and/or homodimer appear to antagonize the biological actions of IL-12. IL-12 p40 transgenic mice developed high serum levels of p40 monomer and the homodimer p80 and exhibited increased susceptibility to intracellular pathogens and reduced delayed-type hypersensitivity responses [63]. Over-expression of p40 in transgenic mice diminished bladder carcinoma eradication [64] by IL-12. Over-production of p40 reduced T_H_1-mediated myoblast allograft rejection [65], but did not prevent the development of a T_H_1-like response in mouse cardiac transplantation [66]. IL-12 p40 was intensely over-expressed by airway epithelium in mice during paramyxoviral bronchitis [46]. Patients with active systemic lupus erythematosis show high serum levels of IL-12 p40 monomer without elevated p70 heterodimer levels [67]. These reports indicate that p40 can be produced discoordinately from complete IL-12 p70 with substantial consequences for immune-inflammatory responses.

Interleukin-23 (IL-23) is a dimer composed of the IL-12 p40 chain and a unique p19 chain that is related to but not identical with IL-12 p35 [68]. IL-23 signals through a dimeric receptor composed of the IL-12R-β1 chain joined to a unique IL23R subunit, and acts through Jak-STAT signaling as does IL-12 [69]. IL-23 induces proliferation of selected T-cell populations and stimulates IFN-γ production [68]. IL-23 is produced by dendritic cells and macrophages, and can be induced by viral infection [70]. It is possible that the over-expression of IL-12 p40 that both we and Huaux [71] observed in mice exposed to silica could be destined for use as a component of IL-23, but IL-23 was not measured in either study. Neither IL-12 nor IL-23 must be critical for the development of silicosis or for the induction of IFN-γ gene expression in mice exposed to silica because IL-12 KO mice lacked p40 but showed both silicosis and IFN-γ mRNA levels comparable to wild-type mice.

### Interleukin-12 in silicosis

Huaux and associates reported persistent over-production of both IL-12 p40 protein and mRNA, but not IL-12 p70, in female NMRI mice treated with quartz silica by IT instillation [71]. They also compared female B6 mice with B6-background IL-12 p35^-/- ^mice that over-expressed p40, and B6-background IL-12 p40^-/- ^mice of the same strain we used for the present report and treated the mice with IT silica at 1 mg or 5 mg per mouse or saline [40]. At the lower dose of silica the extent of silicosis (pathology, OH-proline) was similar in the wild-type and the IL-12 p40^-/- ^mice, paralleling the results we report herein. At the higher dose of silica the IL-12 p40^-/- ^mice developed less fibrosis than the wild-type mice, while the IL-12 p35^-/- ^mice developed more fibrosis than the wild-type mice. When exogenous recombinant IL-12 p40 peptide was administered 30 days after silica instillation, the amount of fibrosis increased in both wild-type and IL-12 p40^-/- ^mice. They proposed that IL-12 p40 over-abundance increased the intensity of the macrophage response to silica and the extent of fibrosis.

Our results and their findings with 1 mg quartz are similar and supportive. Their findings with the 5 mg dose of quartz suggest that the absence of IL-12 p40 may diminish the response to silica at high doses. There are several differences between our two test systems, as well as the similarities noted. Huaux et al used a single IT instillation of silica, while we used 12 days of inhalation exposure. The IT dose could result in less uniform distribution of particles, and may allow larger particles to reach the alveolar spaces than is permitted by aerodynamic filtration of inhaled air. The particle size distribution used for the IT instillation was not reported, while we used quite small particles (Mass Median Aerodynamic Diameter 1.3 μm). Huaux et al exposed mice to quartz silica, while we used cristobalite silica for inhalation. We have reported previously the lung burden of silica retained immediately and at selected times after inhalation of cristobalite [8], while the lung burden following intratracheal quartz was not described. Our last time point was 112 days, while theirs was 60 days. They used female mice, while we used male mice. It is likely that the difference between our results and theirs (less silicosis in IL-12 p40^-/- ^with higher doses of silica) can be explained by these differences in experimental design.

Macrophages respond to a variety of chemotactic signals, such complement C5a, MCP-1 (CCL2), MIP-1α (CCL3), p80, and other mediators. These signals appear to be highly redundant, although each may act in a slightly different manner or at a different stage in the process of macrophage migration. A large literature supports the importance of blocking or deleting one or more of these chemokines in specific diseases. Russell and colleagues showed the importance of IL-12 p80/Rβ1 signaling, but not IL-12 or p40 monomer signaling, for lung macrophage recruitment during murine Sendai virus infection [72]. Huaux and associates [40] showed decreased recruitment of macrophages, neutrophils and lymphocytes following IT instillation of silica into IL-12 p40 KO mice, and attributed the effect to the absence of p80 homodimer. In the present study we did not observe any obvious changes in the abundance of macrophages in the lung lesions of IL-12 KO mice exposed to silica, and the overall extent of the pathology, the lung wet weight, and the increase in collagen were not reduced compared to wild-type mice. Bronchoalveolar lavage was not performed in these studies, and thus pulmonary macrophages were not quantified directly. Our results imply that p80 is not essential for the expression of silicosis, but do not indicate whether or not this chemokine may contribute to macrophage recruitment in silicosis in normal mice.

### IL-18 biology

Interleukin-18 (IL-18) is a member of the IL-1 family that augments the activity of IL-12 in the generation of T_H_1-type immune responses [73]. It cannot act alone to induce IFN-γ production, but rather acts in concert with IL-12 to produce this effect. We surveyed this cytokine because we postulated that it might contribute to the increased IFN-γ production we reported previously [11,12]. IL-18 mRNA abundance was decreased in wild-type mice at early time points following silica exposure, and was at normal levels at 112 days. Like IL-15 or IL-12 p40, and in contrast to IL-12 p35, IL-18 mRNA appeared to be produced constitutively at moderate levels in air-exposed mice of both strains. We examined the abundance of IL-18 message because this cytokine can augment the effects of IL-12, and may be an important signal to bias T_H_1 or T_H_2 polarization [74,75]. It has pleotrophic and sometimes paradoxical effects [75]. As shown in Figure 7, we did not find it up-regulated at an mRNA transcript level. IL-18 is produced as an inactive pro-peptide that must be cleaved by caspace-1 to achieve a biologically active form. Thus, IL-18 is regulated at a post-translational level as well as through transcription. The factors that augment or inhibit IL-18 production are largely unknown. We do not have an explanation for why mRNA abundance is decreased after silica exposure. These observations suggest that IL-18 may not be an important stimulus towards a T_H_1-like response or for IFN-γ production in silicosis. Experiments augmenting, blocking, or deleting IL-18 active protein would be needed in order to test its role directly.

### Mechanisms of silicosis: innate versus adaptive immunity

There appears to be a complex cytokine network through which macrophages, lymphocytes, fibroblasts, and other cell types interact to produce characteristic pathological responses to silica in the lung. While the pivotal function of macrophages in silicosis has been highlighted, the roles that might be played by lymphocytes in this process are less clear [1-7,14]. Brown and associates found increased CD4+ T-cells and a T_H_1-like phenotype following intranasal quartz in New Zealand Mixed mice, a strain prone to autoimmune disease. Hubbard reported no decrease in silicosis in T-cell deficient "nude" (Balb/c^nu/nu^) mice [76], while Barbarin and colleagues observed decreased silicosis in mice treated with anti-CD4 antibody [15].

The initial adaptive immune response to infection or antigens is usually driven towards either a T-helper type 1 (T_H_1) or a T-helper type 2 (T_H_2) phenotype [77,78]. The T_H_1 phenotype dominates the response to intracellular pathogens such as *Mycobacterium tuberculosis *and features intense production of IFN-γ, believed to be driven by IL-12 and enhanced by IL-18 [24,34,79]. The T_H_2 phenotype is featured in responses to parasitic infections and is prominent in allergic responses, with IL-4, IL-5, and IL-13 as abundant cytokines. These two types of responses were believed originally to be mutually exclusive: IFN-γ suppresses T-cell production of IL-4 and drives T-cells towards a T_H_1 phenotype, while IL-4 has the opposite effect. Unfortunately, this categorical classification is often blurred in complex or chronic diseases, and features of both a T_H_1 and a T_H_2 response may be found simultaneously [80]. IFN-γ activates uncommitted NK cells, T-cells, and macrophages, but inhibits fibroblast proliferation and responsiveness to transforming growth factor-beta (TGF-β). IL-4 may also activate uncommitted lymphocytes, and IL-13 enhances fibroblast collagen production driven by TGF-β. Current theory proposes that in the lung T_H_2-like responses appear to enhance fibroblast proliferation and collagen production, while T_H_1-like responses are pro-inflammatory but may be anti-fibrotic [81,82]. The T_H_1/T_H_2 paradigm was derived from analysis of T-lymphocytes participating in an infectious or allergic disease. It is now clear that many cell types in addition to T-cells can produce IL-4, IL-13, IFN-γ, and other relevant cytokines. Macrophages, bronchial epithelial cells, and possibly alveolar Type II pneumocytes may be additional sources for these mediators. How can we apply these theories to the pathogenesis of silicosis and resolve some of the conflicting data regarding the abundance and role of IFN-γ, IL-4 or IL-12?

An alternative, rapid, and more primitive reaction pathway to toxic substances and invading pathogens is the innate immune response [83,84]. It is not antigen-specific, and does not require MHC-restricted antigen presentation. The innate immune response would be a likely pathway for silicosis, since silica is not apparently an antigen, and there is no evidence for an immunospecific response to it. The innate immune response can also involve up-regulation of IFN-γ production by natural killer cells and other lymphocytes through the actions of IL-15 [28,85-87]. IL-15 is critical to the bone marrow proliferation and peripheral organ function of NK cells [28,87-90]. IL-15 participates in lung inflammation, and is essential for the local pulmonary maintenance and activation of NK cells [31,85,91]. Mice that are deficient in IL-15 do not generate mature NK cells, and lack critical innate host defense and tumor defense functions [92]. IL-15 can drive IFN-γ production in NK cells and T-lymphocytes, and provides a signal pathway to bias the innate immune response towards a T_H_-1-like phenotype. We observed substantial IL-15 mRNA in the lung tissues from air-exposed mice and slight up-regulation of IL-15 in silica-exposed, as shown in Figure 8. This finding supports the concept that IL-15 could be an essential driving mechanism in lymphocyte activation and IFN-γ production in silicosis.

Silicosis is not an antigen-driven adaptive immune response to a specific antigen, pathogen, or allergen. It appears to be a chronic innate immune response in which the offending agent, silica, is persistent indefinitely but is not antigenic. T-lymphocytes are not essential, or play a minor amplifying role [76]. Kunkel [81] has proposed that chronic immune-mediated lung diseases follow sequential stages: (1) an initial innate immune response to foreign challenge, (2) an acquired immune stage with first a T_H_1-like response followed by a T_H_2-like response, and finally (3) a repair phase with fibroblast proliferation and collagen deposition. Strieter and Keane [82] have invoked the concept that a T_H_1-like response may favor resolution of injury and inflammation, while a T_H_2-like response may lead to progressive fibrosis. Silicosis may fit squarely within this paradigm. Although there is no evidence for true antigen-specific T_H_1 or T_H_2 response in silicosis, increased levels of IFN-γ, IL-4, and other cytokines may create a T_H_1-like or T_H_2-like local milieu. Because biologically active IL-12 p70 protein does not appear to be either increased or essential (this report, Huaux and colleagues), other mechanisms must be invoked to drive IFN-γ production, NK cell activation, and the aspects of the process that resemble the T_H_1 response. We propose IL-15 as a candidate for this role. The primarily signals that might induce IL-4 or IL-13 in silicosis have not been identified.

We suggest that the innate immune response to silica particles persists and evolves over time. With human exposure over a working lifetime, newly inhaled particles may repeatedly initiate the cycle, while older sequestered material may drive progressive fibrosis. It is likely that the higher the dose the more extensive the initial injury and the more rapidly the responses may cycle through the phases outlined above. With IT instillation of silica the initial phases of macrophage response and T_H_1-like cytokines may pass very quickly, and may only be captured at early time points (note the up-regulation of IFN-γ mRNA found by Hubbard et al [36] at 4 and 24 hours). The chronic fibrotic T_H_2-like phase dominated by IL-4 and IL-13 may be in ascendance very soon after IT exposure, and the T_H_1-like response may no longer be apparent. When rodents are exposed by inhalation a lower dose of silica is applied. This may extend the initial phase of the response by resident macrophages, and may allow the early T_H_1-like response with IFN-γ to continue for at least 4 months, as we have observed. We postulate that in both model systems a transition would occur from the T_H_1- to the T_H_2-like response; the higher the dose of silica, the earlier the transition. These responses could be quite localized within the lung, such that some early silicotic lesions with many recruited mononuclear cells have abundant IFN-γ while more advanced fibrotic lesions are dominated by IL-4. As noted above, T-lymphocytes and NK cells may not be the only sources for these cytokines, and structural lung cells may contribute to the signaling as well.

We view IFN-γ as having an amplifying role on inflammatory cells in the initial phases of silicosis. Macrophages responding to silica and recruited NK cells and T-cells responding to macrophage chemotactins may produce IFN-γ, leading to subsequent further macrophage and lymphocyte activation. In this manner abundant IFN-γ early in the response to silica may exacerbate inflammation and cause a net increase in early collagen deposition, while the absence of IFN-γ may reduce the extent of the initial pathology and lead to less early collagen deposition as we observed [14]. IFN-γ might also have a modulating anti-fibrotic role in the later responses by suppressing fibroblast proliferation and inhibiting IL-4 production. If the initial dose of silica is high, then T_H_2-like responses may dominate from the outset [42].

Because IFN-γ is abundant in mice exposed to silica [11-13], and mice with the IFN-γ gene deleted develop less silicosis than IFN-γ-sufficient mice [14], we believe that IFN-γ is an important mechanistic pathway in silicosis. The pathways leading to IFN-γ production during intracellular pathogen infection and following exposure to antigens that induce a T_H_1-type response favor IL-12 and IL-18 produced by macrophages and other cell types [24,34,79]. This sequence is an important pathway in the adaptive immune response.

## Conclusion

We conclude that the axis of IL-12 driving lymphocyte activation and IFN-γ production, the primary adaptive immune response pathway towards a T_H_-1 phenotype, is not essential for the full manifestations of silicosis in mice exposed to a crystobalite silica aerosol. It is likely that IL-18 is not a major contributor to this response either, since IL-18 mRNA was not increased following silica exposure and IL-18 is not sufficient alone to trigger IFN-γ production [73]. Pathways other than IL-12 and IL-18 appear to be driving lymphocyte activation and recruitment and IFN-γ production in silicosis, with IL-15 a likely candidate. This concept implies that silicosis is a chronic innate immune-inflammatory response that does not convert to adaptive immune response pathways over time. More research is required in order to explore these mechanisms.

## Methods

### Inhalation exposure of mice

Wild-type C57Bl/6 (WT) and Interleukin-12 deficient (IL-12 KO) male mice were purchased from The Jackson Laboratory, Bar Harbor. ME at 6 weeks of age, and were housed in positive-pressure pressure barrier housing units (Thoren, Hazelton, PA) with mouse chow and water provided *ad libitum*. The IL-12 KO mice were developed by Jeanne Magram [33] and were available commercially (Jackson Stock No. 002693; B6.129S1-*Il12b *^*tm1jm*^). The mice were exposed by inhalation in separate chambers for 5 hours per day for 12 days to sham-air, rutile titanium dioxide (TiO_2_, MMAD = 0.5 μm) at 72 mg/m^3 ^± 30 mg/m^3 ^(mean ± SD) or cristobalite silica (MMAD = 1.3 μm) at 61 mg/m^3 ^± 16 mg/m^3^. The chambers utilized horizontal flow temperature- and humidity-conditioned HEPA-filtered air with continuous environmental monitoring and daily gravimetric measurements of ambient dust concentrations. The WT mice were exposed to air or TiO_2 _or silica and, simultaneously, the IL-12 KO mice were exposed to air or silica. The characteristics of the exposure system and the features of silicosis in mice exposed under these conditions have been described in detail [8,93,94].

### Study schedule & organ harvest

Groups of 5 mice were studied for each strain, exposure condition, and time point. Mice were studied 1, 14, 42, and 112 days after the exposure was completed. Mice were killed by intra-peritoneal injection of sodium pentobarbital (200 mg/kg). Two animals in each group had the trachea cannulated, the thorax opened, the inferior vena cava and aorta transected, the right lung cross-clamped at the hilum and the left lung inflated with 4% paraformaldehyde at 10 cm H_2_O pressure, and held for 3 minutes. The isolated right lung was removed, weighed, and frozen immediately for OH-Pro analysis (total lung collagen). The inflated left lung was removed, fixed for 24 hours in 4% paraformaldehyde at room temperature, then transferred to 70% ethanol and held at 5°C. The fixed lung specimens were embedded in paraffin, sectioned at 4 μm, and stained with hematoxylin and eosin (H&E) for microscopic examination. Three mice in each group had the thorax opened immediately, the right lung removed, weighed, and frozen for hydroxyproline analysis, and the left lung removed, weighed, and immediately plunged into liquid nitrogen. The frozen left lung specimens were held at -80°C until mRNA was extracted and analyzed. Thus, in each group there were 5 samples for lung collagen, 3 samples for mRNA abundance, and 2 specimens for histopathology.

### Histopathology

Lung tissue sections stained with H&E were examined by light microscopy at 40- to 400-fold magnification. A semi-quantitative 5-level lung pathology score was used to grade the extent of abnormalities in each microscopic field at 200×. The grading scale is shown in Table 1. The labels of the slides from the lung sections were masked and presented in random order for scoring by one investigator (GSD). Ten microscopic fields with lung parenchyma were scored and averaged to generate a composite score for each mouse. Approximately 1 in 10 of the slides was recycled for repeat scoring to assess intra-observer variation. The average of these two scores was used for mice that were scored twice. Lung tissue sections were stained with Masson's Trichome to reveal fibrillar collagen and connective tissue matrix proteins, and were photographed by light microscopy at 400× original magnification.

### Lung collagen

Total lung collagen for the right lung of each mouse was estimated by measuring the OH-Pro content by colorimetric assay, as previously described [8]. Briefly, the right lung was dried for 24 hours at 60°C, powdered with pestle, and weighed. Half the powder was digested in 6N HCl at 110°C for 18 hours, neutralized with 6N NaOH, reacted with chloramine-T solution, perchloric acid and Ehrlich's reagent, and the optical density of the solution measured at 560 nm. Serial dilutions of trans-hydroxy-L-proline were used to prepare a standard curve for comparison. The results were expressed as μg OH-Pro per right lung.

### Cytokine gene expression

The abundance of mRNA for cytokine genes of interest was assessed using a commercial ribonuclease protection assay (Riboquant gene sets mCK-1b and mCK-2b, BD Biosciences Pharmingen, San Diego, CA). The mRNA from the left lung specimens was extracted in Trizol (Life Technologies, Grand Island, NY) and purified through 100% cold ethanol. The kit mixture of cDNA oligomers was transcribed as anti-sense mRNA with radiolabeling with ^32^P-UTP. Alliquots of lung total RNA were reacted with the labeled mixture of anti-sense mRNA's, digested with RNase, applied to the lanes of a polyacrylamide vertical gel, electrophoresed through tris-borate-EDTA buffer to separate undigested protected fragments, and the gel dried for 1 hour. A film sheet (Kodak, Biomax MR) was applied to the gel and exposed for 18 hours at -80°C for visualization of bands. The gel was then applied to an imaging screen and the density of each band read in a phosphoimager (Bio-Rad, Hercules, CA). The expression of IL-12 p40, IL-12 p35, IL-15, IL-18, and interferon-gamma (IFN-γ) were examined for this report. Probes for L32 and glyceraldehyde-3-phosphate dehyrogenase (GAPDH) were included in the mixture as reference standards for constitutively expressed genes not expected to change in abundance. The phosphoimager density for each cytokine band was adjusted for the abundance of GAPH and L32 in the same lane by averaging the latter two values, dividing the cytokine value by the average, and multiplying the result by 100. The 3 values from the mice in each strain/exposure/time point group were averaged to express a mean value for each cytokine of interest.

### Statistics

Data for all variables were expressed as mean ± SD. Differences among groups due to strain, exposure, or time after exposure were assessed by one-way analysis of variance (ANOVA) with post-hoc testing by the Bonferroni method (Systat v10, SPSS, Inc, Chicago, IL).

## Abbreviations

BALT Bronchial-associated lymphoid tissue

GAPDH Glyceraldehyde-3-phosphate dehydrogenase

H&E Hematoxylene and eosin stain

IFN-γ Interferon-γ

IL-# Interleukin-#

IL-12 KO IL-12 deficient mice (B6.129S1-*Il12b *^*tm1jm*^)

IT Intratracheal instillation

NK-cell Natural killer cell

OH-Pro Hydroxyproline

TGF-β Transforming growth factor-β

TiO_2 _Titanium dioxide

## Competing interests

The authors do not have any financial, academic, or other personal interests that influence or compete with the results and opinions presented in this report.

## Authors' contributions

GSD directed the research, examined the pathology, analyzed the data and was primarily responsible for writing the manuscript. LMP designed the laboratory experiments, developed the methods, and performed the laboratory analysis. MR assisted with the design of the experiments, analysis of the data, and preparation of the manuscript. DRH performed the inhalation toxicology and supervised the animal care. All authors read and approved the final manuscript.
